# Prediction of nutritive sucking in preterm babies (<34 weeks) and preterm sucking readiness scale

**DOI:** 10.1186/s40748-019-0113-9

**Published:** 2019-11-04

**Authors:** Nisha Kumari, Ashish Jain, Siddarth Ramji

**Affiliations:** 1 Department of Pediatrics, Maulana Azad Medical College, New Delhi, 110002 India; 2 Department of Neonatology, Maulana Azad Medical College, New Delhi, 110002 India

**Keywords:** Nutritive-sucking, Preterm, Infant, Preterm sucking readiness scale

## Abstract

**Objective:**

To determine predictors of nutritive-sucking in babies < 34 weeks and estimate the appropriate preterm sucking readiness (PTSR) score as an indicator of readiness of nutritive-sucking.

**Methods:**

Prospective longitudinal observational study conducted in Neonatal unit of a referral hospital attached to Medical College. Forty-nine inborn babies of 28-34 weeks’ gestation and on full gavage feeds were enrolled.

**Results:**

(a) Nutritive-sucking was achieved at a median age of 14 days (Range 7–50). (b) Low birth weight (LBW) (< 1531.1 ± 142.8) and lesser gestational age (GA) (< 32.8 ± 1) were poor predictors (*p* < 0.05) and have a significant independent negative association (Correlation birth weight (BW) - 0.0222, GA − 2.2177) with age at which established nutritive-sucking was achieved. (c) PTSR score of ≥9 had the best prediction for achievement of nutritive-sucking at 14-days of life, with a sensitivity of 92.3% and specificity of 100%.

**Conclusion:**

PTSR score is a sensitive and specific tool to predict the readiness for nutritive-sucking in preterm babies < 34 weeks.

## Introduction

Many infant factors such as gestational age (GA) at birth and the birth weight, intrauterine growth status, illness severity, respiratory support, gender and maternal factors such as maternal education, number of other children, socioeconomic status, education, age of mother, breastfeeding experience, mode of delivery, antenatal visits, influence the development of feeding skills and the length of feeding progression [1, 2]. Feeding disorder clinics have reported feeding disorders in greater than 40% of babies that were former preterm babies [3]. Yet, there are no simple tools to aid healthcare professionals to decide when to initiate breastfeeding in these babies. Suck:Swallow ratio can vary during breastfeeding, a suck-swallow ratio of 2:1–4:1 was present in the groups with preterm infants [4]. Ratio is increased as feeding is progressed [5]. During bottle feeding the ratio tends to be more consistent at 1:1 [6]. We have taken suck:swallow 2:1 ratio as standard to compare the nutritive sucking in the preterm babies. The respiration was not taken into account in the ratio in our study [3]. However, it is difficult to assess the suck and swallow simultaneously in real time by examining the baby. Thus, we decided to assess a simple objective tool called Preterm Sucking Readiness (PTSR) Score [7], particularly since there were no other studies that had validated this tool since its original publication. We also proposed to determine an appropriate PTSR score that would be indicative of nutritive sucking in preterms 28 to 34 weeks of gestation.

## Material and methods

This was a prospective longitudinal observational study done in a Neonatal unit of a tertiary medical college hospital in North India over a 9 month period (May 2015–January 2016).

Inborn babies admitted to the NICU were eligible for enrolment if they were born between 28 and 34 weeks of gestational age and are on full gavage feeding**. Those with** encephalopathy (any grade according to Levene’s classification), major congenital malformation, oro-nasal malformation, receiving any respiratory support or sedative drugs, and had undergone any surgery were excluded.

The primary outcome was “Nutritive sucking” defined as a suck to swallow ratio of 2:1 over a 3-min observation of feeding on an empty breast assessed by videography at 7, 14, 21 and/or 28 days after enrollment. There were no prior studies done to evaluate PTSR scale (Appendix), therefore sample size estimation was done as per pilot study, it was decided a-priori to enrol 40 eligible babies in the study. In all enrolled babies maternal characteristics (age, socioeconomic status (using modified Kuppuswamy scale) [8], education, parity, relevant medical and obstetric history, and mode of delivery) were recorded. Neonatal characteristics of the enrolled babies (gestational age, birth weight, intrauterine growth status (Fenton’s growth chart) [9], Clinical Risk Index for Babies [CRIB] score [10], morbidities and feeding details) was also recorded.

Assessment for sucking Readiness**:** All enrolled babies were assessed by a single investigator (who was trained prior to starting the study in doing PTSR) once every day at a fixed feeding session for their sucking readiness while sucking on an empty breast (non-nutritive sucking) for 3-min using the PTSR. We have used the original PTSR scale. Physiological parameters (Respiratory rate, Saturation) were recorded for the safety of neonate before assessing PTSR score. Continuous monitoring of oxygen saturation was done during the entire period of feeding. No scores were given for the physiological parameters. Thereafter, the baby was assigned a score which was a sum total of score of behavioural state just prior to feeding, score of transition between behavioural state during handling/breastfeeding and score of feeding readiness behaviour (sucking, rooting, mouthing and showing interest at the breast). As soon as the babies attained an established nutritive sucking, they were no longer assessed. No additional support was given to the enrolled mothers. The standard NICU protocols were followed.

Videographic-recording. First video recording of feeding session was performed on day 7 of enrolment and thereafter weekly. However, neonates were also considered for assessing nutritive sucking earlier than the scheduled weekly assessment if there was an appreciable increase in the daily PTSR score. This however was in addition to the scheduled weekly assessment for nutritive sucking. Video graphic recording of the feeding by the baby on the breast were done using a high quality Nikon Coolpix L840, 16 megapixel camera. However, neonates were also considered for assessing nutritive sucking earlier than the scheduled weekly assessment if there was an appreciable increase in the daily PTSR score. This however was in addition to the scheduled weekly assessment for nutritive sucking. The dynamic video-graph frame ensured the capture of the maternal breast (to monitor latching of the baby), baby’s mouth and neck (to monitor sucking and swallowing). Based on these observation the suck:swallow ratio was estimated during play back of the videorecording, first the number of sucks were counted and then video was replayed to count number of swallows, and thus suck:swallow ratio was calculated independently by two of the investigators.

### Statistical analysis

The predictors of nutritive sucking at 7, 14, 21 and 28 days were assessed by univariate analysis. Multivariate analysis (ANNOVA) was done for significant variables. Categorical data were compared by Chi Square/Fisher exact test. Sensitivity and Specificity of PTSR score was done using receiver operating characteristic curve (ROC) analysis. A *p*-value of 0.05 was taken as significant.

## Results

The study enrolled 49 preterm babies, of whom 41(83.6%) completed the study. 7 babies expired and 1 went LAMA (leave against medical advice) before completion of the study. Of the 49 babies enrolled, 20 were a result of twin pregnancy and 5 a result of triplet pregnancy. Only 1 mother had eclampsia. Table 1 summarizes the characteristics of the enrolled babies.

**Table 1:** Baseline characteristics of study participants

Maternal characteristics (*n* = 49)	Estimates
Age of mother (yrs) [mean(SD)]	26.5 (5.8)
Socioeconomic status	
Lower middle n(%)	40 (18.4)
Upper lower n(%)	9 (81.6)
Primigravidan(%)	24 (49.0)
Singleton n(%)	24 (49.0)
Antenatal visits ≥3 (%)	38 (77.5)
Mode of delivery	
Vaginal (%)	43 (87.8)
LSCS (%)	6 (12.2)
Neonatal characteristics, (*n* = 49)	
Gestational age (weeks) [mean(SD)]	32.2 (4.7)
Birth weight (g) [mean(SD)]	1418.1 (288.9)
Gender	
Male (%)	21 (42.9)
Intrauterine growth status	
AGA (%)	45 (91.8)
SGA (%)	4 (8.2)
CRIB score [mean(SD)]	1.4 (1.9)
Illness	
RDS (%)	2 (4.1)
Sepsis (%)	2 (4.1)
Hyperbilirubinemia (%)	1 (2.1)
Respiratory support therapy^a^	
IPPV (%)	3 (6.1)
CPAP (%)	7 (14.2)
Supplemental oxygen (%)	18 (36.7)
Age at starting of enteral feeding (Days) [mean(SD)]	0.9 (1.9)
Median (Range)	0 (0–10)
Age at parentral fluids discontinuation (Days) Median (Range)	0 (0–15)
Weight at discontinuation of parentral fluids (g) [mean(SD)]	1385.2 (245)

*LSCS* Lower Segment Cesarean Section, *AGA* Appropriate for Gestational Age, *SGA* Small for Gestational Age, *CRIB* Clinical Risk Index for Babies, *RDS* Respiratory Distress Syndrome, *CPAP* Continuous Positive Airway Pressure

^a^ The respiratory support received by the study subjects was prior to enrollment

**Fig. 1:**
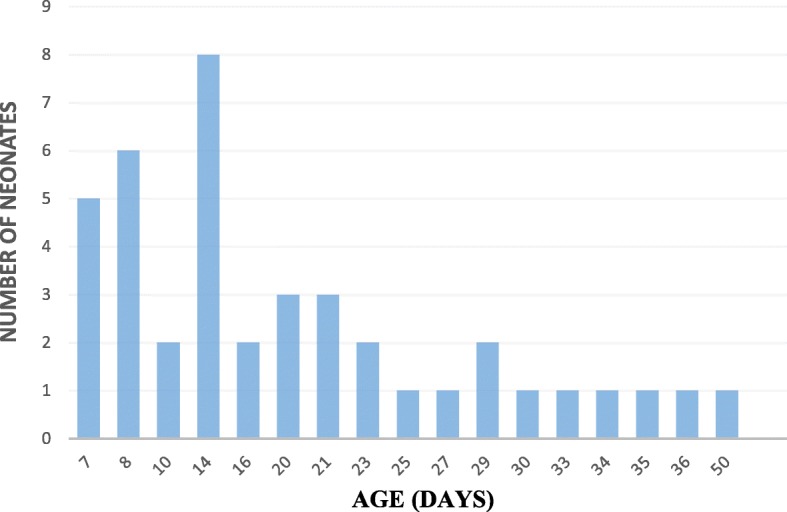
Frequency distribution of age (days) at establishment of nutritive sucking

The authors conclude that nutritive sucking was achieved at a median gestational age (GA) of 14 days (range 7–50 days) (Fig. 1). Mean GA of the participants was 32 weeks, median time to achieve nutritive sucking was 14 days. Even though, it may be debated that this may be the maturity due to the corrected gestational age of 34 weeks, when the babies should feed, prior studies and the WHO guidelines states that gestation age at birth determines the ability and not the corrected maturational gestation [11]. Table 2 depicts the association of predictor variables and age of achievement of nutritive sucking. Babies with higher gestational age, birth-weight and lower illness severity (lower CRIB score) achieved nutritive sucking significantly earlier. On multivariate analysis it was observed that gestation (*p* = 0.049) and birth weight (*p* = 0.003) had a significant independent negative association with age at achievement of nutritive sucking. There was variation between two observers in only 4 videos.

**Table 2:** Association of predictor variables and age when nutritive sucking achieved

	Age nutritive sucking achieved				
Predictor variables	Within 7 days (*n* = 5)	Within 8–14 days (*n* = 16)	Within 15–21 days (*n* = 8)	After 21 days (*n* = 12)	*p* *value
Age of mother (yrs) [mean(SD)]	26.4 (5.0)	25.8 (3.6)	25.8 (2.7)	26.5 (6.1)	> 0.05
Lower-middle socio-economic status (%)	2 (40)	1 (6.2)	2 (25)	3 (25)	> 0.05
Primigravida (%)	2 (40)	9 (56.2)	2 (25)	8 (66.7)	> 0.05
Singleton (%)	5 (100)	8 (50)	4 (50)	1 (8.3)	> 0.05
Gestational age (weeks) [mean(SD)]	33.2 (0.4)	32.8 (1.0)	32.6 (1.2)	30.8 (1.5)	< 0.05
Birth weight (g) [mean(SD)]	1561 (138.9)	1531.1 (142.8)	1438.8 (165.2)	1211.8 (201.2)	< 0.05
Intrauterine growth retardation (SGA) (%)	0	0	0	3 (23.1)	> 0.05
CRIB [mean(SD)]	0 (0)	0.9 (1.4)	1.3 (2.1)	2.8 (2.3)	< 0.05
Median (range)	0 (0–0)	0 (0–4)	0 (0–5)	2 (0–6)	
Breastfed previous children (%)	4 (80)	8 (50)	5 (62)	4 (33.3)	> 0.05

*CRIB* Clinical Risk Index for Babies, *SGA* Small for Gestational Age

* *p* value by comparing all the groups

**Fig. 2:**
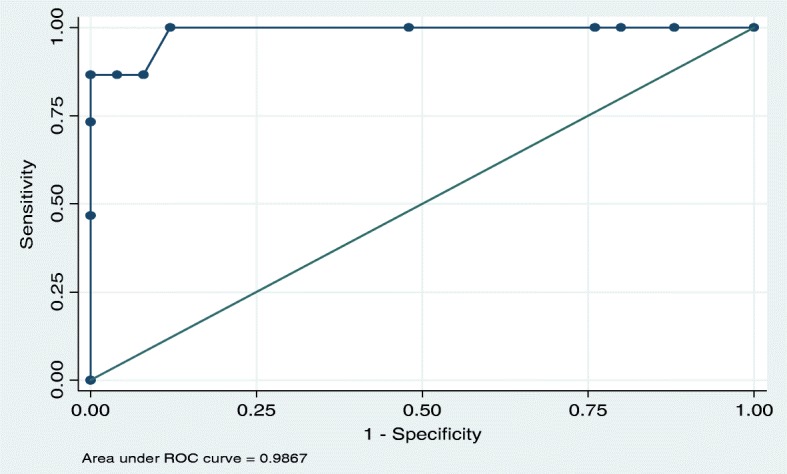
ROC analysis of PTSR score on day 6 for predicting nutritive sucking at Day 7

**Fig. 3:**
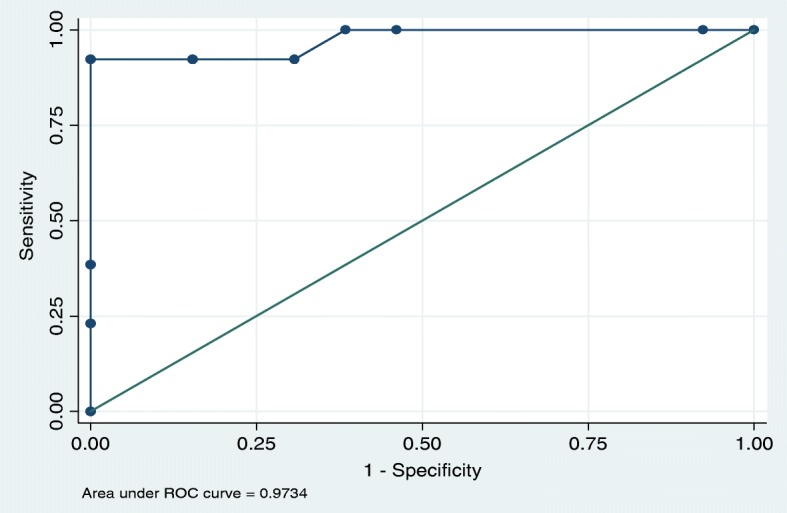
ROC analysis of PTSR score on day 13 for predicting nutritive sucking at Day 14

It was observed that a PTSR score of > 9 had the best predictive score for attainment of nutritive sucking. For achievement of nutritive sucking by day 7, a PTSR score of > 9 had a sensitivity of 86.7% and specificity of 100.0%. Similarly, for achievement of nutritive sucking by day 14 (*n* = 26), a PTSR > 9 had a sensitivity of 92.3% and specificity of 100%. (Figs. 2, 3).

## Discussion

In the present study nutritive sucking was achieved at a median age of 14 days amongst preterm babies 28–34 weeks of gestation. Birth weight and gestation had a negative independent association with the age at achievement of nutritive sucking. A PTSR score of 9 or more had a high sensitivity and specificity in identifying achievement of nutritive sucking in this population of preterm babies.

As was noted in the present study, Pickler et al. [12] and White-Traut et al. [13] also reported that older GA at birth was a significant predictor of higher frequency of feeding readiness behaviours. Birth-weight was another significant predictor in the present study, which was consistent with the findings reported by White-Traut et al. [13]. This may be because higher birth-weight is related to improved coordination of breathing, sucking and swallowing, thus leading to better feeding as observed by Reynolds et al. [14]. The infant’s medical condition also influences the transition from gavage to full oral feedings Bazyk [15] et al. and Dodrill et al. [16]. The present study also found that illness severity (CRIB Score) is directly proportional to the time required for transition from tube to breastfeeding on univariate analysis, but not on multivariate analysis.

Other tools which are available are either too complex or time consuming, such as Neonatal Oral-Motor Assessment Scale (NOMAS) [17] contains separated 13 characteristics of jaw movement and 13 characteristics of tongue. NOMAS is not a reliable tool as the inter-rater agreement with respect to the diagnosis was moderate to substantial. There is a debate regarding the validity of NOMAS when used in preterms as this scale was developed from term babies [18]. Preterm Infant Breastfeeding Behavior Scale (PIBBS) [19] developed based on the observations of preterms from 30 to 36 weeks of gestation during a breastfeeding session at anytime during the day. Six items were assessed with a score being attributed to each item. There was acceptable agreement between the observer but lower agreement between mothers and observers. In our study we studied behavioural state and the effect of handling on it along with infant feeding behaviour in babies of 28–34 weeks gestational age. We had done weekly videographic recording of the breastfeeding session which was reviewed by two independent observer for excluding interrater bias. When serially assessed in < 34 weeks babies a PTSR score of ≥9 indicates readiness to nutritive sucking. However, this tool needs validation with a larger sample size; this would enable its use in day to day practice in NICUs by the care givers.

Limitation of this study is that the sample size was less. The results of the study may not be extrapolatable to very preterm babies < 30 weeks of gestation since the sample size in that gestational strata was less. Further studies with larger sample size are needed before widespread clinical application of PTSR scale. Inclusion of a high proportion of subjects from twin or triplet pregnancies greatly reduces the biodiversity of the population.

## Conclusion

- The gestational age and birth weight are important factors affecting achievement of nutritive sucking compared to many other factors including sickness of babies.
- PTSR score can be used as an objective, simple and important adjuvant in optimization and early initiation of breastfeeds in LBW babies.

## Figures and Tables

**Table 4:**
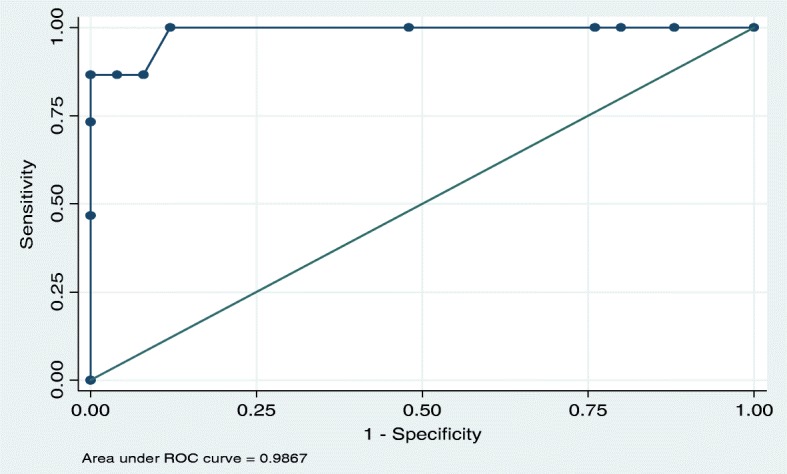
Feeding Readiness Behaviour

## Data Availability

An electronic search was carried out using PubMed, Cochrane library, and Google scholar to collect data. Search was limited to literature and studies published in English language. Key-words used during search were Nutritive-sucking, preterm, infant, Preterm Sucking Readiness Scale.

## Appendix

### Preterm Sucking Readiness Scale (PTSR)

**Table 3:** Physiological assessment

S.No	Variable	Method of assessment	Scoring
1.	Respiratory rate	Baseline RR counted before feeding session 30–40 breaths/minute	Yes = 1. No = 2
2.	Oxygen saturation	1. Baseline saturation > 90%	Yes = 1. No = 2
		2. Direction of change	increase =1, decrease =2
		3. <=5–10% difference from baseline	Yes = 1. No = 2
		4. Maximum and minimum SpO_2_ during handling	Max. Min.
